# In Memoriam

**Published:** 1873-08

**Authors:** L. B. Anderson

**Affiliations:** Hanover, Virginia


### IN MEMORIAM.

---

HANOVER, VIRGINIA, July 8, 1873.

*Editors Southern Medical Record:*

The papers of to-day bring the sad intelligence of the death of one of your Associates, Dr. ALBERT TERRELL, in the city of Richmond. Dr. TERRELL was about forty years of age, and died in the enjoyment of the Christian's hope. He commenced the practice of medicine about 1850, with a limited preparatory education and moderate talents. But by close application, laborious study, accurate observation, kindness, gentleness, amiability, sobriety, an honorable and manly deportment, and a Christian's life, he made rapid progress in his profession, and acquired an enviable reputation and an extensive practice. A successful practitioner, a worthy citizen, a warm-hearted friend, an affectionate husband, a kind father, and an humble Christian, he has gone to his reward, and left a vacuum which may not soon be filled.

Yours respectfully,

L. B. ANDERSON.

We deeply regret to learn of the decease of our Associate, Dr. TERRELL. May his manly virtues and noble life be kept green in the memory of his brethren. We also thank Dr. Anderson for the notice above.

